# Regenerative potential of tonsil mesenchymal stem cells on surgical cutaneous defect

**DOI:** 10.1038/s41419-017-0248-4

**Published:** 2018-02-07

**Authors:** Sung-Chan Shin, Yoojin Seo, Hee Young Park, Da-Woon Jung, Tae-Hoon Shin, Haejin Son, Young Keum Kim, Jin-Choon Lee, Eui-Suk Sung, Jeon Yeob Jang, Hyung-Sik Kim, Byung-Joo Lee

**Affiliations:** 1 Department of Otorhinolaryngology-Head and Neck Surgery, Biomedical Research Institute, Pusan National University School of Medicine, Pusan National University Hospital, Busan, Republic of Korea; 2 Biomedical Research Institute, Pusan National University School of Medicine, Pusan National University Hospital, Busan, Republic of Korea; 3 Department of Pathology, Biomedical Research Institute, Pusan National University School of Medicine, Pusan National University Hospital, Busan, Republic of Korea; 4 Department of Otorhinolaryngology-Head and Neck Surgery, Biomedical Research Institute, Pusan National University School of Medicine, Yangsan Pusan National University Hospital, Yangsan, Republic of Korea; 5 Department of Otorhinolaryngology-Head and Neck Surgery, Ajou University School of Medicine, Suwon, Republic of Korea

## Abstract

As tissue engineering and regenerative medicine have evolved recently, stem cell therapy has been investigated in the field of impaired wound healing. Several studies have reported that mesenchymal stem cells derived from various tissues including bone marrow and adipose tissue can exert the regenerative efficacy in the wound healing. Previously, we have demonstrated the isolation and characterization of tonsil-derived mesenchymal stem cells (TMSCs) with excellent proliferative property. In the present study, we aimed to evaluate the regenerative efficacy of TMSCs in the wound healing process. Two distinct cutaneous surgical defects were generated in the dorsum of mice. Each wound was treated with TMSCs or phosphate-buffered saline (PBS), respectively. After sacrifice, the skin and subcutaneous tissues around the surgical defect were harvested and assessed for inflammation, re-epithelialization, dermal regeneration, and granulation tissue formation. The administration of TMSCs into wound beds significantly promoted the repair of surgical defects in mice. Especially, TMSCs efficiently contributed to the attenuation of excessive inflammation in the surgical lesion, as well as the augmentation of epidermal and dermal regeneration. To elucidate the underlying mechanisms, TMSCs were analyzed for their potency in immunomodulatory ability on immune cells, stimulatory effect on the proliferation of keratinocytes, and fibroblasts, as well as the regulation of fibroblast differentiation. TMSCs inhibited the non-specific or T-cell-specific proliferation of peripheral blood mononuclear cells, as well as the M1 polarization of macrophage-like cells. Moreover, TMSCs augmented the proliferation of skin-constituting fibroblasts and keratinocytes while they suppressed the differentiation of fibroblasts into myofibroblasts. Taken together, our findings demonstrate the regenerative potential of TMSCs in wound healing process through the regulation on inflammation, proliferation, and remodeling of various skin cells, implying that TMSCs can be a promising alternative for wound repair.

## Introduction

Wound healing process is initiated in response to various deleterious stimuli to restore the tissue homeostasis as well as to limit further damage^1,2^. It is a highly organized, well-controlled procedure which consists of somewhat overlapping but specific stages: inflammation, proliferation, and maturation/remodeling^3^. Several types of cells including tissue resident- or recruited immune cells, keratinocytes, tissue stem cells, and fibroblasts are involved in wound repair process^1,4^. Adequate environmental cues originated from various extracellular matrix and soluble signaling molecules are also essential for a successful wound repair^5,6^. Importantly, failure in any single phase of the normal wound response can lead to chronic wound, resulting in delayed recovery and, even worse, the permanent loss of tissue. In the era of accelerated population aging with the growing risk of chronic or progressive medical conditions such as diabetes, the chronic wound has become a significant economic burden to the society^7,8^. Although some surgical techniques including skin graft and flaps have been tried to treat the delayed- or non-healing wound in combination with general symptomatic management (e.g. anti-inflammatory agent and pain reducer)^9,10^, the therapeutic outcome is often limited with low functional recovery score. Given that disrupted cellular homeostasis, including persistent inflammation, excessive fibrosis, and decreased angiogenesis underlies chronic wound formation, more comprehensive approaches are required for complete wound repair.

As a variety of studies have been performed in the field of tissue engineering and regenerative medicine recently, stem cell therapy has been widely applied to intractable diseases to overcome the limitation of conventional treatment options^11^. In particular, mesenchymal stem cells (MSCs), which possess less ethical- and safety issues than embryonic stem cells or induced pluripotent stem cells, can not only maintain their self-renewal capacity but also differentiate into multiple cell types^12,13^. Furthermore, through the interaction with other types of cells and microenvironment, MSCs can actively migrate into inflammatory or damaged sites to secrete a number of paracrine factors, including growth factors and cytokines associated with regeneration, immunomodulation, and angiogenesis^14–16^. Because of these beneficial characteristics, therapeutic potential of MSCs has been investigated and proven for chronic wound repair. Several groups have reported that bone marrow-derived MSCs (BMSCs) and adipose tissue-derived MSCs can stimulate neo-angiogenesis and reduce excessive inflammation to improve cutaneous wound healing^17–20^. These findings support the advantages of MSC therapy against wound healing impairment; however, the therapeutic efficacy and relevant undelying mechanisms of MSCs from different sources have not been fully uncovered and the harvesting of MSCs from bone marrow and adipose tissues, the main sources of adult MSCs, is somewhat invasive.

Tonsillectomy, a surgical procedure to remove the tonsil in case of chronic tonsillitis or tonsillar hypertrophy, is one of the most common surgery in the field of otolaryngology. Interestingly, several groups including our own group have successfully isolated palatine tonsil-derived MSCs (TMSCs) and further revealed that TMSCs exhibit similar characteristics with MSCs from other tissues in their morphology, the pattern of surface marker expression, as well as the potential of proliferation and differentiation^21,22^. Furthermore, growing evidence has suggested the therapeutic benefits of TMSC application in various diseases such as liver fibrosis, allergic rhinitis, and peripheral nerve injury^23–25^. In our own previous studies, we successfully isolated and characterized TMSCs and further elucidated their unique functions represented by superior proliferation potential, indicating that TMSCs might be a promising source for the large-scale manufacturing of therapeutics from stem cells or their derivatives^21,26^. To investigate the possible beneficial efficacy of TMSCs in wound repair and to further elucidate their underlying mechanisms, we created excisional wound splinting model and delivered TMSCs into wound beds.

## Results

### TMSCs accelerate wound healing in vivo through the suppression of inflammation and the induction of regeneration

**Fig. 1:**
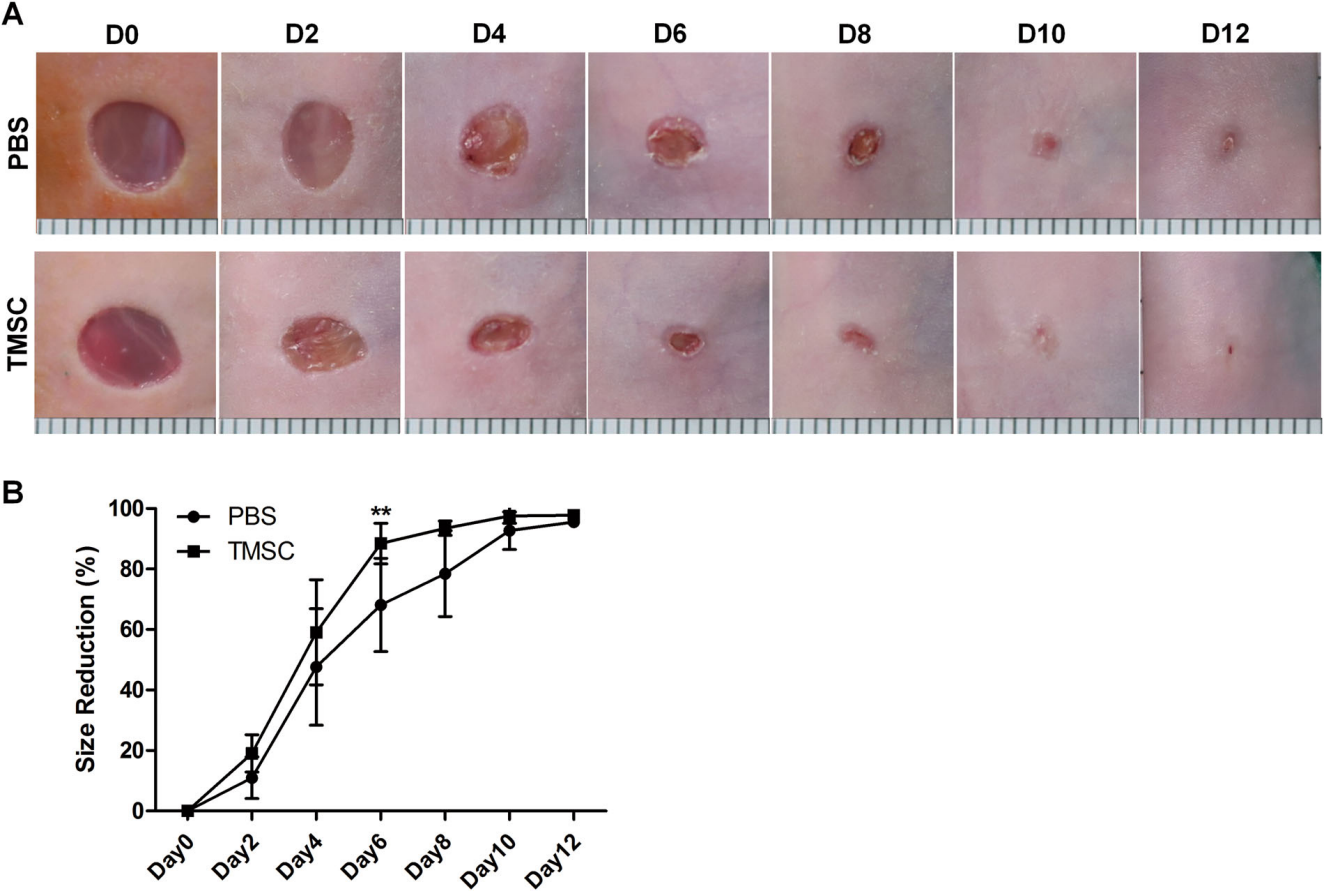
Efficacy of TMSCs in punch biopsy wound model. **a** Representative time-course images of wound closure in a murine excisional skin wound model after TMSCs or PBS delivery. **b** The reduced wound size (%) relative to day 0 lesion (0%; complete wound closure is considered as 100%) was determined every other day for 12 days. ** *P* < 0.01. Results are shown as mean ± SD

We first investigated whether the transplantation of TMSCs could demonstrate any beneficial effects against excisional wound splinting model. Based on our previous results demonstrating the optimal dosage and delivery route of MSCs from various sources against murine models of several disorders including skin inflammatory disease, we infused one million cells locally onto wound beds to expect optimum efficacy^27–30^. Therefore, one million TMSCs were placed on the wound beds after wound generation and the reduction rate of wound area was measured every other day until day 12. PBS was placed onto the wound beds in the opposite side of TMSC-treated wound as a vehicle control group. Wound areas diminished time-dependently in both PBS- or TMSC-treated groups and TMSCs promoted the wound closure throughout the monitoring period (Fig. 1a, b). Particularly, TMSCs significantly accelerated the reduction of wound area on day 6, compared to PBS-treated group (Fig. 1a, b).

**Fig. 2:**
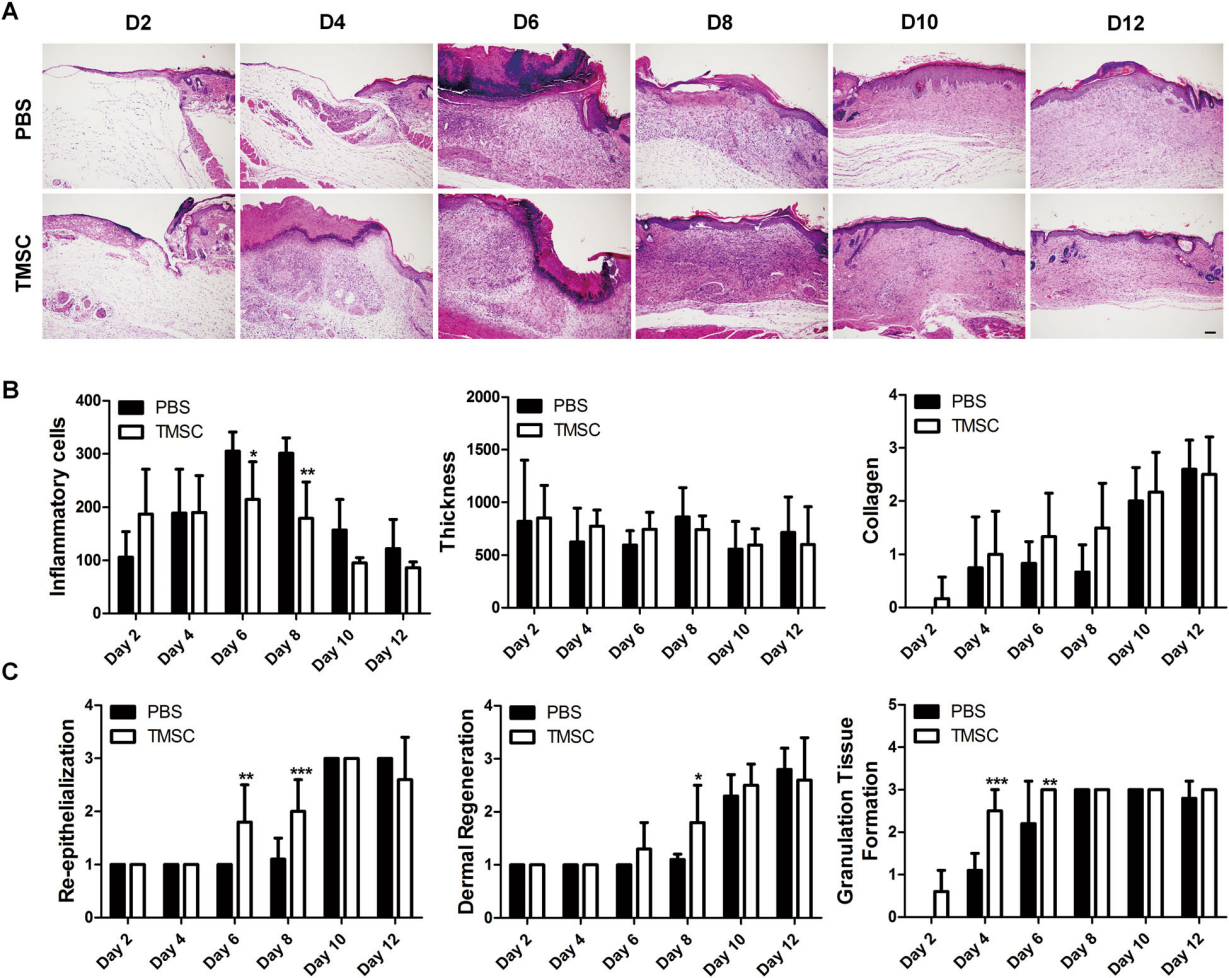
Histopathological analysis of wound lesion after TMSC transplantation. **a**–**c** Skin wound lesions were collected and processed for H&E staining to perform the histological assessment. **a** Representative H&E- stained sections are shown. **b**,**c** Wound healing efficacy of TMSCs compared to PBS was evaluated based on wound-associated indicators including inflammatory cell recruitment, epidermal thickness/regeneration, collagen deposition, re-epithelialization, and granulation tissue formation. TMSCs reduced local inflammation as well as tissue granulation and enhanced epidermal regeneration during the middle stage of wound healing (days 4–8). *P < 0.05, **P < 0.01, ***P < 0.001. Scale bar = 200 μm. Results are shown as mean ± SD

Histopathological evaluation based on hematoxylin and eosin (H&E) staining demonstrated consistent efficacy of TMSC treatment. On days 6 and 8, TMSCs significantly downregulated the infiltration of inflammatory cells, including neutrophils, lymphocytes, histocytes, and plasma cells, compared to PBS-treated group (Fig. 2a, b and Supplementary Figure S1A-B). Moreover, TMSCs significantly attenuated the infiltration of inflammatory cells producing tumor necrosis factor (TNF)-α on days 6 and 8, while they slightly enhanced the production of interleukin (IL)-10, a prominent anti-inflammatory cytokine (Supplementary Figure S2A and B). Epidermal thickness and collagen deposition were not significantly different between two groups (Fig. 2a, b). Interestingly, epidermal regeneration was significantly improved by TMSC treatment at days 6 and 8 (Fig. 2a, c). Moreover, TMSC transplantation not only induced dermal regeneration significantly at day 8, but also augmented the formation of granulation tissue at days 4 and 6 (Fig. 2a, c).

When the efficacy of TMSC treatment was compared to that of adipose tissue-derived MSCs (AMSCs), MSCs from both sources exerted similar potency of therapeutic efficacy in wound repair (Supplementary Figure S3A and B). We next verified the efficacy of conditioned media harvested from TMSCs (TMSC CM) to assess the function of soluble factors without the transplantation of cells. However, the topical application of TMSC CM did not exhibit any supportive or therapeutic function in wound healing process (Supplementary Figure S3C and D).

**Fig. 3:**
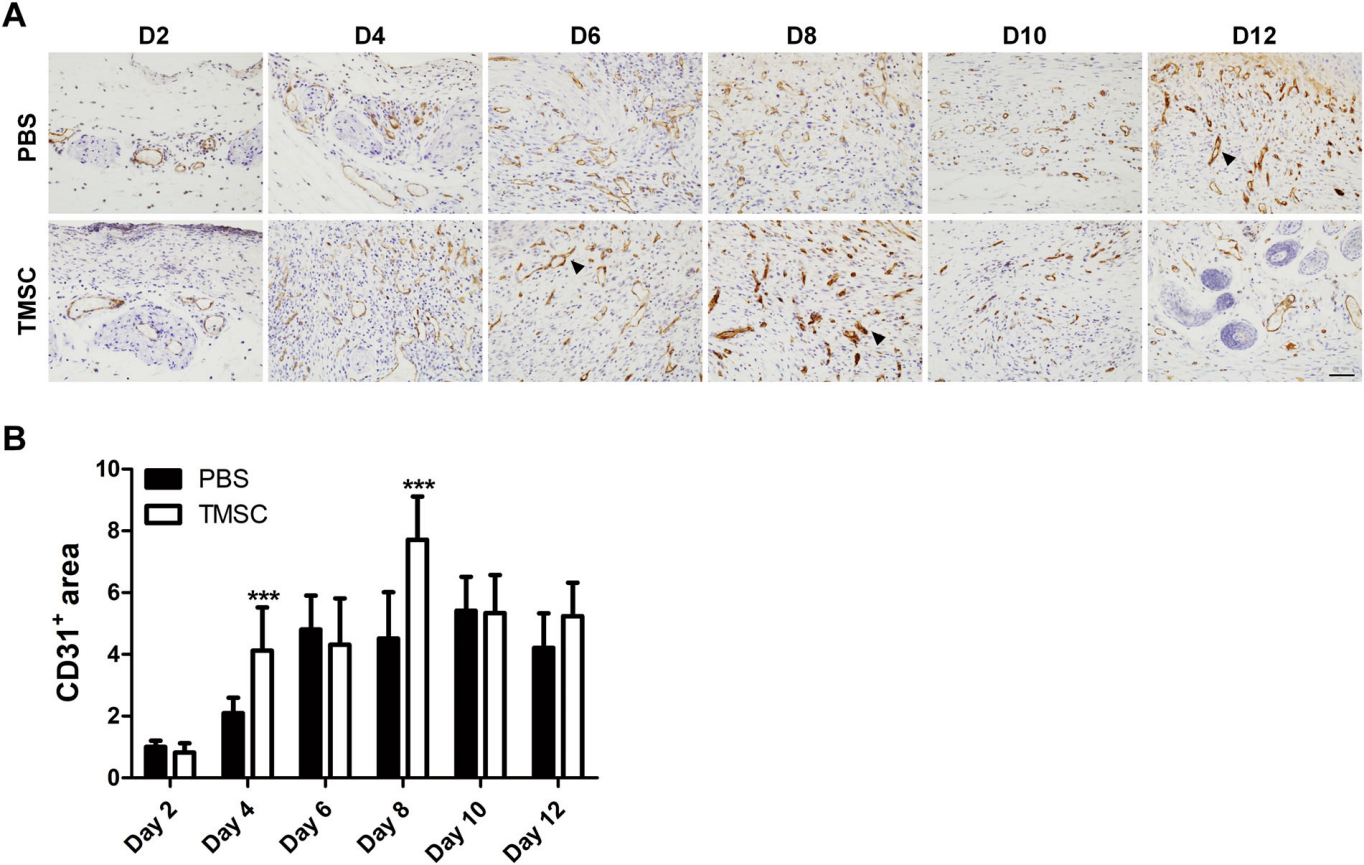
Angiogenic function of TMSCs in the wound healing process. **a**, **b** To evaluate the pro-angiogenic impact of TMSCs on wound healing, formalin-fixed skin sections were labeled with endothelial specific marker, CD31. **a** The distinctive sign of neovascularization was observed during the healing process in both groups. Representative CD31^+^ cells are indicated by arrowheads. **b** Relative comparison of CD31^+^ area between TMSCs and PBS-treated wound showed that angiogenesis occurred more actively in the TMSCs group than in the PBS group. ***P < 0.001. Scale bar = 100 μm. Results are shown as mean ± SD

Given that angiogenesis is critical in tissue regeneration as well as wound repair^31^, we next investigated whether TMSC treatment could affect the neovascularization during wound healing process. Skin sections containing wound beds were stained with anti-CD31 antibodies and CD31^+^ vasculatures were quantified by measuring stained area. Compared with PBS-treated wounds, neovascularization was significantly increased in TMSC-treated wounds at days 4 and 8 (Fig. 3a, b).

Taken together, these results indicate that TMSC treatment can enhance wound repair in gross and histopathological evaluation via immunomodulation and regeneration, presumably mediated by paracrine function of transplanted cells in the wound beds.

### TMSCs direct macrophages toward anti-inflammatory type and suppress the proliferation of inflammatory cells

**Fig. 4:**
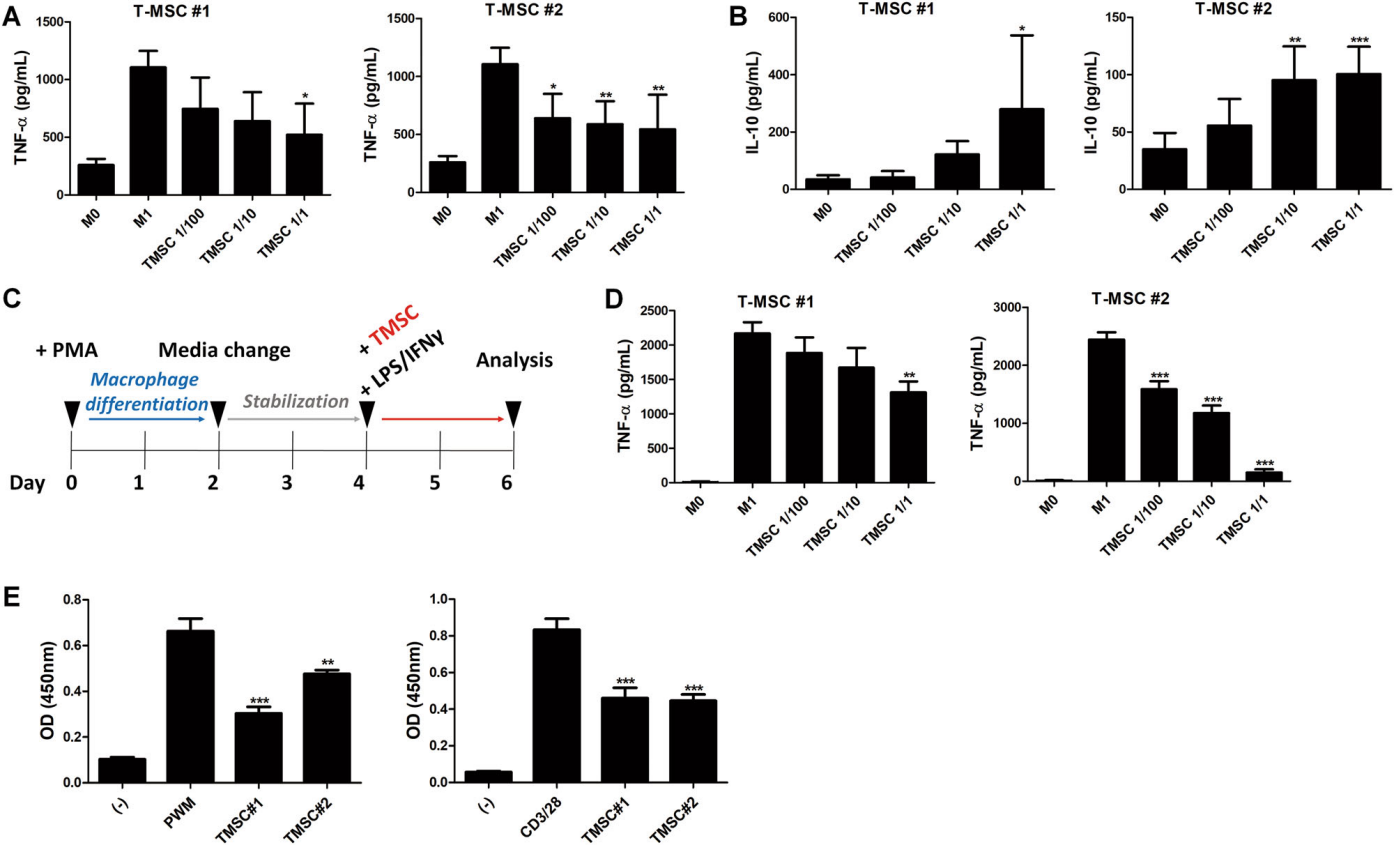
Immunosuppressive effects of TMSCs on activation of macrophage-like cells and proliferation of inflammatory cells. **a-b** Mouse BMDMs were cu-cultured with TMSCs and the production of cytokines representing macrophage polarization was determined by ELISA. **a** The secretion of TNF-α was measured for the analysis of M1 polarization. **b** IL-10 production was measured for the analysis of M2 polarization. **c** Outline of macrophage-like cell differentiation from THP-1 and co-culture protocol with TMSCs. **d** THP-1-derived macrophage-like cells were activated by LPS/IFN-γ and co-cultured with TMSCs isolated from two different donors for 2 days at different ratios. The secretion of TNF-α was measured by ELISA. **e** The proliferation of PBMCs stimulated with PWM or antibodies for CD3/28 was evaluated by BrdU incorporation assay. *P < 0.05, **P < 0.01, ***P < 0.001. Results are one representative experiment of three or the cumulative of three independent experiments. Results are shown as mean ± SD

The first stage of wound healing is the inflammatory phase. This phase begins immediately after wound generation to prevent the infection as well as the loss of blood and fluid. After 2–3 days, monocytes migrate to wound area and differentiate into macrophages, key regulatory cells for later event in wound repair^32,33^. Therefore, we then assessed the functional alteration in macrophages after co-culture with TMSCs. Mouse bone marrow-derived macrophages (BMDMs) were polarized in the presence of TMSCs. Activation of macrophage-like cells into M1 type with lipopolysaccharide (LPS) and interferon (IFN)-γ led to the secretion of tumor necrosis factor (TNF)-α (Fig. 4a). TMSC addition inhibited TNF-α secretion from M1 type macrophage-like cells in a dose-dependent manner (Fig. 4a). More interestingly, co-culture with TMSCs induced the maturation of BMDMs without any activation into IL-10 secreting M2 type (Fig. 4b). This regulatory function of TMSCs were consistently observed in TMSCs from two different donors. To prove this regulatory function of TMSCs on macrophage polarization in human macrophages, macrophage-like cells were matured from THP-1, followed by stabilization and activation in the presence of TMSCs (Fig. 4c). As observed in co-culture experiments using mouse BMDMs, TMSCs demonstrated the similar dose-dependent suppressive ability on the production of TNF-α by M1 type macrophage-like cells (Fig. 4d). However, TMSCs did not significantly augment the secretion of IL-10 by macrophage-like cells without any stimulation for polarization (data not shown).

Because in vivo TMSC transplantation attenuated the infiltration of inflammatory cells, we next explored the effect of TMSCs on the proliferation of inflammatory cells using peripheral blood mononuclear cells (PBMCs). All TMSCs isolated from different donors significantly suppressed the proliferation of PBMCs or T lymphocytes, stimulated with Poke weed mitogen (PWM) or antibodies for CD3 and CD28, respectively (Fig. 4c, d).

Taken together, our findings demonstrate that TMSCs can create inflammatory milieu beneficial for wound repair by the suppression of macrophage polarization into inflammatory types as well as the proliferation of various inflammatory cells.

### TMSCs support dermal regeneration through the augmentation of fibroblast proliferation and migration

**Fig. 5:**
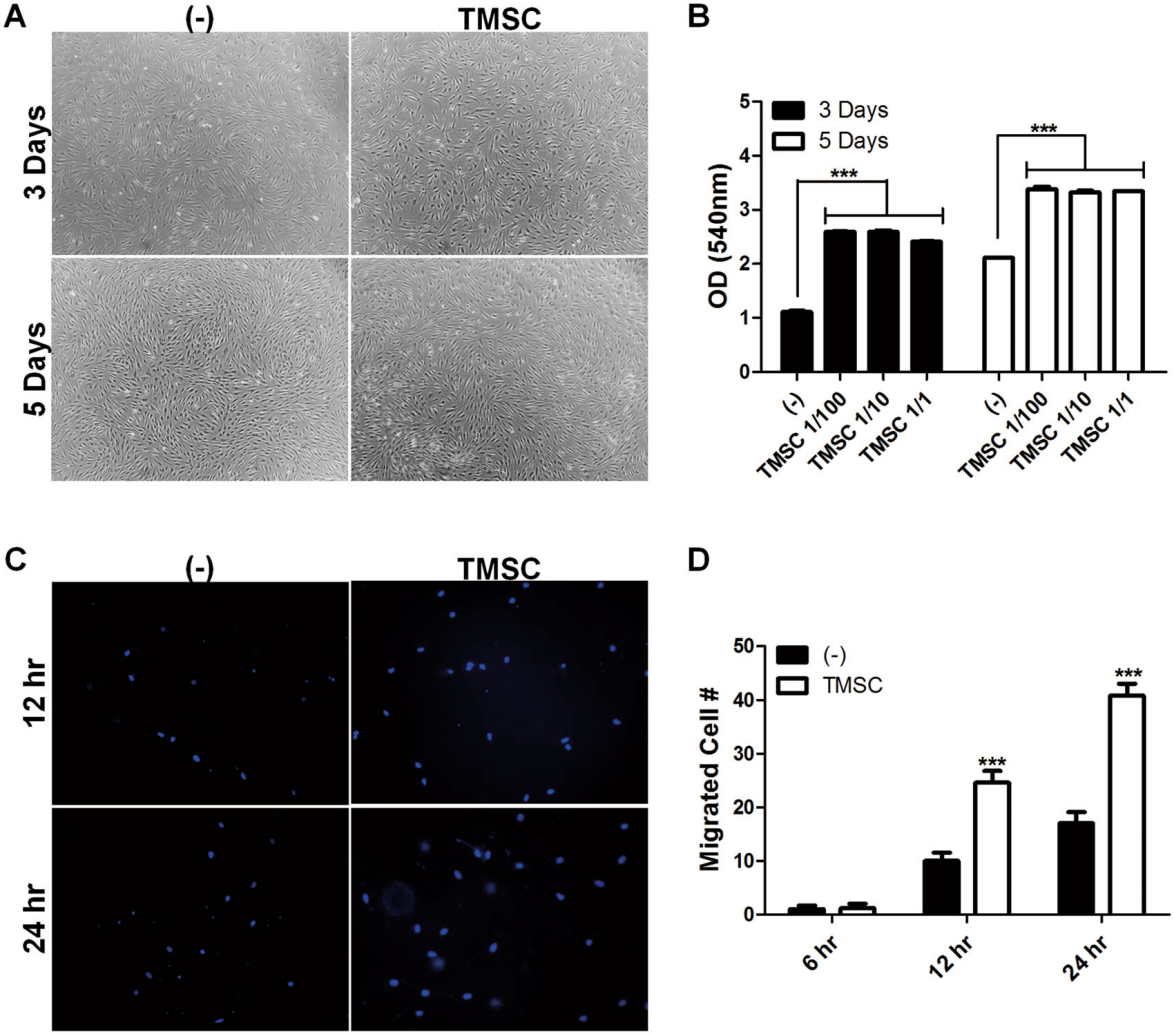
Regulation of dermal fibroblast proliferation and migration by TMSCs. **a-b** After indirect co-culture using transwell, the proliferation of human dermal fibroblasts was analyzed by **a** observing phase contrast morphology and **b** determining MTT activity. **c-d** TMSCs were plated into lower chambers of the transwell system and then fibroblasts were seeded into upper chambers, subsequently incubated for 6, 12, and 24 h. Migration of fibroblasts were determined by DAPI staining. **c** Representative fluorescent images of migrated fibroblasts were shown and **d** the total number of migrated cells were quantified. ***P < 0.001. All results are presented as mean ± SD from at least three independent experiments

The second stage of wound repair is the proliferation phase which begins on the third day after injury and lasts for about 2 weeks. This phase is characterized by cellular proliferation and migration, deposition of extracellular matrix, granulation tissue formation along with angiogenesis^33^. In our previous results obtained from in vivo experiments, TMSC transplantation onto wound beds critically contributed to dermal regeneration at day 8, which is included in the proliferation phase. Therefore, we sought to investigate whether TMSCs can affect the proliferation and migration of fibroblasts, which constitute dermis. TMSCs were co-cultured with human dermal fibroblasts using transwell system to prevent direct cell-to-cell contact within the range of 1:100 to 1:1, based on the TMSC:fibroblast ratio. The proliferation of fibroblasts was significantly increased when they were co-cultured TMSCs from two different donors, regardless of the TMSC:fibroblast ratio (Fig. 5a, b). To determine the chemotactic function of TMSCs to recruit fibroblasts toward wound area, migration assay was performed using transwell. Fibroblasts plated in upper chamber migrated toward TMSCs in lower chamber (Fig. 5c, d).

Our data propose that TMSCs might contribute to dermal regeneration via the stimulation of fibroblast proliferation and the recruitment of fibroblasts into close proximity with wound region.

### TMSCs suppress differentiation of fibroblasts into myofibroblast

**Fig. 6:**
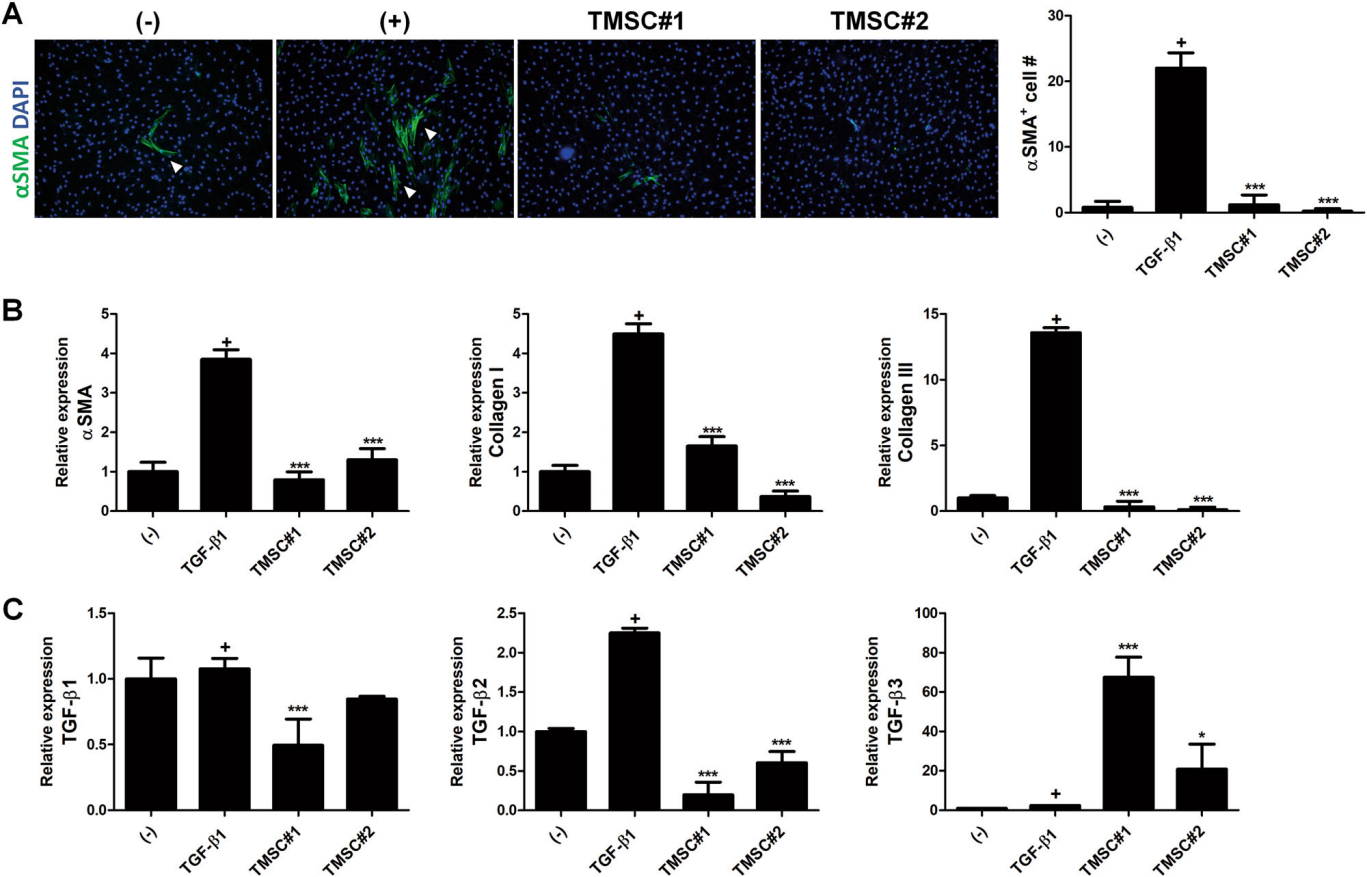
Inhibition of myofibroblast differentiation by TMSCs. **a**–**d** Human dermal fibroblasts were seeded on lower chambers of transwell plates and stimulated with TGF-β1 to differentiate into myofibroblasts, followed by incubation for 2 days with or without TMSCs from two different donors in upper chambers. **a** The expression of α-SMA, a specific marker for myofibroblast, was determined by immunocytochemistry. Representative α-SMA^+^ cells are indicated by arrowheads. **b** mRNA expression of collagen or TGF-β subtypes was analyzed by qPCR. *P < 0.05, **P< 0.01, ***P < 0.001. Results are shown as mean ± SD

In the final stage of wound repair, the remodeling phase, dermal fibroblasts newly generate dermal tissue to remodel wound matrix. After exposure to transforming growth factor (TGF)-β1 in the wound area, fibroblasts differentiate into myofibroblasts. These cells produce excessive amount of extracellular matrix, leading to scar tissue formation and poor regeneration^34^. Therefore, regulatory function of TMSCs on myofibroblast differentiation was analyzed. Differentiation of fibroblasts into myofibroblasts was induced by TGF-β1 treatment and was detected by α-smooth muscle actin (SMA) expression in protein level using immunofluorescence staining. While TGF-β1 treatment increased the number of α-SMA-expressing myofibroblasts, TMSC co-culture significantly abrogated the generation of myofibroblasts (Fig. 6a). Expression of collagen or TGF-β subtypes in mRNA level was further determined by qPCR. Consistently with immunofluorescence staining, α-SMA expression in mRNA level was significantly decreased by TMSC co-culture (Fig. 6b). Moreover, relative expressions of type 1 collagen and type 3 collagen were downregulated by TMSC addition (Fig. 6b). The TGF-β superfamily has been reported to be critically involved in scar formation and TGF-β3 is known to be more profibrotic than TGF-β1 and -β2^35^. Among TGF-β subtypes, relative expressions of TGF-β1 and -β2 were diminished by TMSC co-culture whereas TGF-β3 expression was elevated (Fig. 6c). TMSCs isolated from two different donors exhibited similar potency in the regulation of gene or protein expression in myofibroblasts (Fig. 6a–c).

Our findings imply that TMSCs can inhibit myofibroblast differentiation and regulate gene expression pattern, presumably in a way beneficial for wound repair with complete regeneration.

### TMSCs increase keratinocyte proliferation to promote epidermal regeneration

**Fig. 7:**
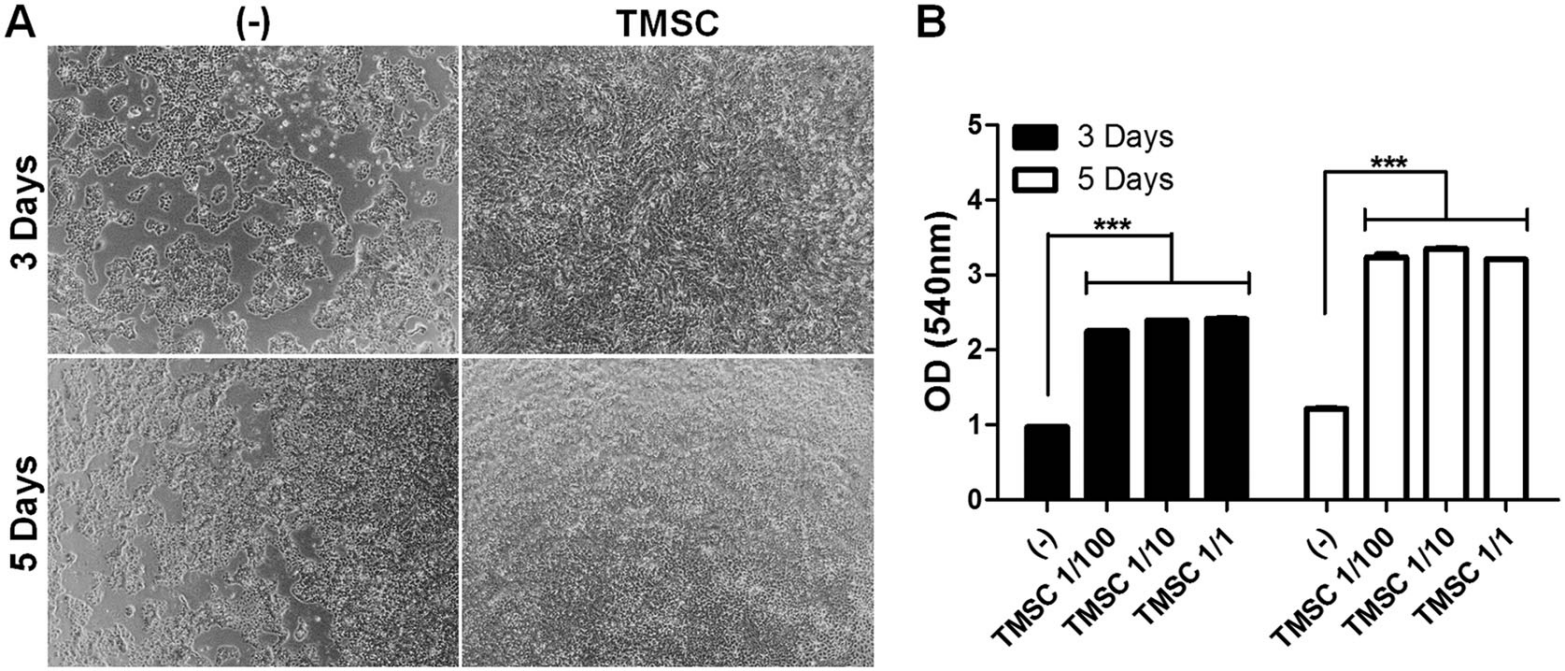
Enhancement of keratinocyte proliferation by TMSCs. **a-b** Human keratinocytes (HaCaT cells) were plated into lower chambers of the transwell system, and incubated for 3 and 5 days in the presence or absence of TMSCs in the lower chamber. **a** Representative phage-contrast images of keratinocytes at days 3 and 5. **b** MTT assay was performed for determining proliferation of keratinocytes at three different ratios. Data are one representative experiment of three or the cumulative of three independent experiments. *** *P* < 0.001. Results are shown as mean ± SD

Keratinocytes are key players in epidermal regeneration. HaCaT cell, an immortalized cell line of human keratinocyte, was co-cultured with TMSCs using transwell to allow paracrine-mediated cell-to-cell interaction without direct adhesion and HaCaT cell proliferation was assessed. TMSCs significantly increased the proliferation of HaCaT cells, regardless of the TMSC:HaCaT cell ratio (Fig. 7a, b). These results demonstrate the stimulatory function of TMSCs on keratinocyte proliferation, indicating that TMSCs might accelerate re-epithelialization in wound repair.

## Discussion

In the present study, we demonstrate for the first time that human TMSC transplantation can accelerate wound repair through immunomodulation and regeneration of dermis and epidermis in the murine excisional splint model. Several previous studies have reported wound healing efficacy of MSCs and MSC-derived conditioned media or exosome, isolated from various human sources, including bone marrow^36^, adipose tissue^37^, and umbilical cord^38–40^. Shin and Peterson^36^ showed that human BMSC grafts enhanced wound healing in murine model of excisional splint wound. Another study from Heo et al.^37^ proved that conditioned media from activated human AMSCs promoted cutaneous wound healing through paracrine mechanisms^37^. Other groups have reported that human umbilical cord-derived MSCs could enhance wound repair when cell itself^38^, conditioned media^39^ or exosome^40^ were delivered onto wound beds. Our findings are consistent with these previous studies. We proved here that TMSC transplantation onto wound beds significantly accelerated the reduction of wound size. Although TMSCs could not demonstrate superior efficacy when compared to AMSCs in murine wound model, TMSCs possess advantages in that they are highly proliferative and more resistant to senescence, as demonstrated in our previous study^21,26,41^. The clinical application of MSCs requires large-scale production of cells for allogeneic therapy because of the limitation in obtaining sufficient quantity of autologous stem cells. Therefore, TMSC could be a promising candidate for both autologous and allogeneic cell therapy.

MSCs have been reported to regulate all three phases of wound repair: the inflammation, proliferation, and remodeling phases. During the transition from the inflammation phase to the proliferation phase, wound state should not regress to chronic inflammation. In this transition period, macrophages resident in wound region play a crucial role to shift inflammation toward regeneration. A number of studies have demonstrated that MSCs could directly or indirectly attenuate inflammatory responses. In our previous study, we found that that MSCs from another source, the umbilical cord blood (CBSC), exhibited macrophage-modulating ability to polarize macrophages toward M2 type, the anti-inflammatory and regenerative type of activated macrophages^41^. In the present study, we proved that TMSCs also possessed similar regulatory function on macrophage polarization. TMSCs significantly downregulated the TNF-α production from equivalent number of M1 macrophages. Inflammatory cells other than macrophages, including T lymphocytes, B lymphocyte, and mast cells, are involved in the progress of skin inflammation and wound repair. In this study, TMSCs impaired the proliferation of PBMCs activated with non-specific mitogen, as well as the proliferation of T lymphocytes activated with antibodies for CD3 and CD28 on T-cell surface. Moreover, in our previous studies, we showed that CBSCs could not only induce the generation of regulatory T cells^29^, but also suppress the activation of mast cells^28^ or B cells^27^, both in vitro and in vivo using relevant immune cells or immune-related disease models. One might envision that TMSCs could possess similar immunomodulatory functions via multiple mechanisms, which makes TMSCs as a promising candidate for wound therapeutics.

Recently, several experimental evidences have proven that MSCs could support the proliferation and remodeling phases to promote wound healing via their regenerative potential. Although some studies suggest that MSCs can directly participate in structure regeneration during wound repair by trans-differentiation into keratinocyte, endothelial cells, and fibroblasts^42–44^, the majority of researchers put greater emphasis on paracrine factor-mediated regeneration of MSCs than direct differentiation. For instance, studies from these groups propose that fibroblasts, keratinocytes, and endothelial cells are affected by MSC-mediated paracrine signaling, resulting in the functional alteration of their proliferation, survival, and migration^45^. In the present study, TMSCs augmented the epidermal and dermal regeneration in vivo, as well as the migration of fibroblasts and the proliferation of both fibroblasts and keratinocytes in vitro through the paracrine-mediated interaction. Moreover, paracrine factors from MSCs are reported to provide anti-scarring effect in wound repair by balancing the expression of TGF-β1, -β2 and -β3^46,47^. Consistent with these reports, we demonstrated here that TMSCs could prevent the differentiation of fibroblasts into myofibroblasts and regulate the balance in expression of TGF-β superfamily during differentiation, elevating the expression of anti-fibrotic TGF-β3. During the proliferation phase of wound repair, angiogenesis is an essential process to provide sufficient nutrients for new tissue forming cells, including fibroblasts^48^. One might doubt that inhibition of TGF-β1 expression in fibroblasts by TMSCs can lead to the decrease in cellular proliferation, because crucial role of TGF- β1 in fibroblast proliferation has been reported by several groups including the recent one by Xiao et al.^49^. However, studies from other groups demonstrated that MSCs can induce the proliferation of fibroblasts through the secretion of various growth factors including basic fibroblast growth factor (bFGF)^50,51^. In the present study, although TMSCs downregulated the mRNA level of TGF- β1 in fibroblast, TMSCs augmented the proliferation of fibroblasts, presumably through the secretion of a variety of growth factors. Lack of adequate microvascularization can result in non-healing chronic wound. MSCs have been reported to secrete paracrine factors to promote the generation of microvascular network and vascular stability^52,53^. In this study, TMSCs exhibited significant angiogenic potential in proliferation phase of wound repair. Since lots of paracrine factors from MSCs such as matrix metalloproteinases (MMPs), tissue inhibitors of MMP, epidermal growth factors, bFGFs, vascular endothelial growth factors, and anti-inflammatory soluble factors are reported to promote wound healing, future studies elucidating the unique paracrine function of TMSCs compared to MSCs from other sources could uncover novel mechanisms of TMSC-mediated wound repair and further suggest the additional advantages of TMSCs as therapeutics. In addition, further studies are required to explore the detailed conditions for MSC treatment including dosage, number and interval of multiple cell administration, as well as the optimization of procedure for cell maintenance and harvesting. Lastly, pre-clinical and clinical references reporting the adverse effects of MSC treatment should be accumulated to strengthen the proof for its safety.

The present study has its limitations in construct validity of animal model for wound repair. Nude mice cannot exactly mimic the process of wound repair in human. In particular, this model might exhibit different mechanisms in the inflammatory phase, because nude mice lack certain population of immune cells, including T lymphocytes. However, Dandekar et al.^54^ reported that the number and function of macrophages were conserved and even limited population of extrathymically matured T cells were observed in athymic nude mice. Given that macrophages are key immune cells in the regulation of the inflammatory phase in wound repair, the model used in this study can partially recapitulate the repair process in human wound. Another limitation also resides in the animal model, in that the model cannot fully describe all three phases of wound repair independently. Therefore, it is difficult to elucidate the exact mechanisms of TMSC efficacy in each phase.

In conclusion, our present study comprehensively revealed the efficacy of human TMSCs in wound repair, as well as the underlying functions of TMSCs for each phase of wound repair. TMSCs efficiently contributed to immunoregulation and regeneration through the interaction with a variety of skin constituting cells and immune cells, indicating that TMSCs might be a promising therapeutic alternative for wound repair.

## Materials and methods

### Isolation and culture of TMSCs

TMSCs were isolated and maintained as described previously^41^. Briefly, TMSCs were isolated from excised palatine tonsil tissue obtained after tonsillectomy. Tonsil tissue was extensively washed with phosphate-buffered saline (PBS), followed by the digestion with 0.075% collagenase type I (Sigma, St. Louis, MO) for 30 min at 37 °C. The pellet was obtained by centrifugation at 1200 *g* for 10 min and was filtered through a 100 μm nylon mesh. Suspended cells were incubated overnight in α-minimum essential media containing 10% fetal bovine serum (FBS) at 37 °C with 5% CO_2_. Non-adherent cells were removed by extensive washing with PBS and adherent cells were maintained and sub-cultured. All procedures using human tonsil tissue or tissue-derived cells were conducted in accordance with guidelines approved by the Pusan National University Hospital Institutional Review Board.

### Wound healing model and TMSC administration

BALB/c nude mice (female, 8 weeks old) were obtained from Samtako (Osan, Republic of Korea) and group housed under specific pathogenic-free conditions in the animal facility of the Pusan National University Hospital. All experiments were approved by and followed the regulations of the Institute of Laboratory Animal Resources (No. PNU-2008-0001), Pusan National University Hospital. The excisional wound model was generated and TMSCs were subcutaneously transplanted. Briefly, after the aseptical preparation of surgical site including hair removal, two 6-mm full thickness skin wounds were created on each side of the dorsal part using a biopsy punch. One million TMSCs suspended in 20 μL PBS were placed onto wound beds immediately, whereas wounds on the opposite side received PBS as vehicle control groups. All wounds were covered with Tegaderm Film (3M, Minneapolis, MN).

### Gross evaluation

Digital photographs of wounds were taken at days 0, 2, 4, 6, 8, 10, and 12. Wound area was measured by tracing the margin of wounds. The percentage of wound size reduction was calculated as the following: % wound size reduction = (area_i_−area_t_)/area_i_ × 100, where area_i_ designates the initial wound area and area_t_ designates the area after time interval.

### Histopathological evaluation

At days 2, 4, 6, 8, 10, and 12, mice were sacrificed and wound beds were excised, fixed in 4% paraformaldehyde followed by consecutive tissue processing steps and embedding in paraffin. Sections of 4 μm thickness were prepared and stained with H&E or anti-CD31 antibody (Abcam, Cambridge, UK). Infiltration of inflammatory cells was measured by counting the number of neutrophils, lymphocytes, histocytes, and plasma cells in tissue sections stained with H&E. Epidermal thickness and collagen deposition were measured. Histological parameters including re-epithelialization (1: minimal epidermal regeneration <50%, 2: moderate epidermal regeneration ≥50%, 3: complete epidermal regeneration = 100%), dermal regeneration, and granulation tissue formation were calculated. Angiogenesis was assessed by measuring the area of CD31^+^ vasculatures using ImageJ software. Histological evaluation was conducted using randomly selected fields from skin tissue sections.

### Co-culture of TMSCs with activated mouse BMDMs and human macrophage-like cells

BMDMs were isolated from the femur and tibia of C57BL/6 mice by flushing with PBS. Harvested cells were resuspended in RPMI-1640 (Gibco BRL, Grand Island, NY) containing 1% l-glutamine, 10% FBS, and 10 ng/mL macrophage colony-stimulating factor and maintained for 7 days at 37 °C and 5% CO_2_ with medium change every 3–4 days. On day 7, BMDMs without any treatment were co-cultured with TMSCs within the range of 1:100 to 1:1 based on the TMSC/M1 ratio. For M1 activation of BMDMs, 100 ng/mL LPS and 50 ng/mL IFNγ were treated along with the addition of TMSCs. Co-culture was maintained for 2 days and culture media was harvested and determined for mouse TNF-α or IL-10 production using commercial enzyme-linked immunosorbent assay (ELISA) kits (R&D Systems, Minneapolis, MN). To generate human macrophage-like cells, THP-1 cells (2.5 × 10^5^/mL) were treated with 200 nM phorbol 12-myristate 13-acetate (PMA, Sigma-Aldrich) for 48 h, followed by the stabilization of PMA-treated cells for additional 2 days in fresh media without PMA. To induce the activation of macrophage-like cells into M1 type macrophage-like cells, LPS (1 μL/mL) and IFN-γ (20 ng/mL) were treated with TMSC addition for co-culture. Cells were incubated for 2 days and culture media was harvested and determined for human TNF-α and IL-10 production using ELISA kits (R&D Systems).

### Proliferation assay of PBMCs and T lymphocytes

TMSCs were treated with mitomycin C (25 mg/mL) at 37 °C for 1 h to inhibit cell proliferation. The cells were plated in 96-well plates at 1 × 10^4^/well. Six hours later, human PBMCs (1 × 10^5^/well in 100 μL media; Zen-bio, Triangle Park, NC) were added with the treatment of PWM (Sigma-Aldrich) or antibodies for CD3 and CD28 (e-bioscience, San Diego, CA), for the activation of mononuclear cells (MNCs) or T lymphocytes, respectively. After 3 days of a co-culture, MNC or T lymphocyte proliferation was determined by a cell proliferation ELISA, bromodeoxyuridine kit (Roche, Indianapolis, IN).

### Proliferation assay of fibroblasts and keratinocytes

Human fibroblasts or keratinocytes (HaCaT cells) were seeded onto the lower chamber of a 12-well transwell plate (0.4 μm pore size) at 5 × 10^4^/well in Dulbecco's modified Eagle's medium media (Invitrogen, Carlsbad, CA) containing 2% FBS. After 6 h of stabilization, TMSCs at different number (5 × 10^4^, 5 × 10^3^, and 5 × 10^2^/well) were added onto the upper chamber. At day 3 and day 5, the proliferation of fibroblasts or keratinocytes was measured by methyl thiazolyl blue tetrazolium bromide (MTT; Sigma-Aldrich).

### Cell migration assay

Fibroblast migration toward TMSCs was determined using transwell with 5 μm pore size. TMSCs (1 × 10^4^/well) were plated onto the lower chamber, followed by incubation for 6 h. Human fibroblasts were seeded onto the upper chamber at 1 × 10^4^/well. After 6, 12, and 24 h of incubation, cells in the upper chamber were fixed with 10% formaldehyde for 10 min, followed by washing with PBS. Using cotton swabs, cells which had not migrated through the pore were removed. Migrated cells were stained with DAPI. After washing with PBS, stained cells were counted using a fluorescent microscope (Leica Microsystems, Wetzlar, Germany).

### Induction and determination of myofibroblast differentiation

Fibroblasts (5 × 10^4^/well) were plated on lower chambers of 12-well transwell plate (0.4 μm pore size). After 6 h of incubation, 5 ng/mL of recombinant human TFG-β1 was treated for 48 h at the presence or absence of TMSCs (5 × 10^4^/well) in upper chambers. The fibroblasts were subsequently subjected to immonofluorescent staining for α-SMA (Abcam) or RNA isolation using TRIzol Reagent (Invitrogen). The number of cells expressing α-SMA was determined from 10 fields of each image. Quantitative real-time-PCR (qRT-PCR) was performed by mixing cDNA synthesized from isolated RNA with primers and SYBR Green PCR Mater Mix (Applied Biosystems, Foster City, CA) using an Real-time-PCR system (ABI7500, Applied Biosystems). The primer sequences used were as follows; α-SMA, GCTACTCCTTCGTGACCACAG (forward) and GCCGTCGCCATCTCGTTCT (reverse); Collagen I, CCTCAAGAGAAGGCTCACGATGGTG (forward) and AGGTCTCACCAGTCTCCATGTTGCA (reverse); Collagen III, GCTCTGCTTCATCCCACTATTA (forward) and TGCGAGTCCTCCTACTGCTAC (reverse); TGF-β1, AGTTGTGCGGCAGTGGTTGA (forward) and GCCATGAATGGTGGCCAGGT (reverse); TGF-β2, TAGACATGCCGCCCTTCTTCC (forward) and AGCACCTGGGACTGTCTGGA (reverse); TGF-β3, AGCACCTGGGACTGTCTGGA (forward) and CAATGTAGAGGGGGCGCACA (reverse).

### Statistical analysis

The mean values of the different groups were expressed as the mean ± SD. All statistical comparisons were made using one or two-way ANOVA followed by the Bonferroni post hoc test for multi-group comparisons using the GraphPad Prism version 5.01 (GraphPad Software, San Diego, CA). Statistical significance designated as asterisks is indicated in the figure legends.

## Electronic supplementary material

Supplemental material
