# Supplementary material for: Regenerative potential of tonsil mesenchymal stem cells on surgical cutaneous defect
Source: Cell Death Dis. 2018 Feb 7;9(2):183. doi: 10.1038/s41419-017-0248-4 (PMC5833728; doi:10.1038/s41419-017-0248-4)
Supplement: Supplementary file 1 — Supplemental material [file 41419_2017_248_MOESM1_ESM.docx]

**Supplementary Information for: Shin *et al*.**

**Regenerative Potential of Tonsil Mesenchymal Stem Cells on Surgical Cutaneous Defect**

Sung-Chan Shin^1*^, Yoojin Seo^2*^, Hee Young Park^1^, Da-Woon Jung^1^, Tae-Hoon Shin^2^, Haejin Son^2^, Young Keum Kim^3^, Jin-Choon Lee^4^, Eui-Suk Sung ^4^, Jeon Yeob Jang^5^, Hyung-Sik Kim^2†^, Byung-Joo Lee^1†^

^1^Department of Otorhinolaryngology-Head and Neck Surgery, Biomedical Research Institute, Pusan National University School of Medicine, Pusan National University Hospital, Busan, Republic of Korea

^2^Biomedical Research Institute, Pusan National University School of Medicine, Pusan National University Hospital, Busan, Republic of Korea

^3^Department of Pathology, Biomedical Research Institute, Pusan National University School of Medicine, Pusan National University Hospital, Busan, Republic of Korea

^4^Department of Otorhinolaryngology-Head and Neck Surgery, Biomedical Research Institute, Pusan National University School of Medicine, Yangsan Pusan National University Hospital, Yangsan, Republic of Korea

^5^Department of Otorhinolaryngology-Head and Neck Surgery, Ajou University School of Medicine, Suwon, Republic of Korea

^*^These authors contributed equally to this work

^†^Authors share co-corresponding authorship

**Running Title: Role of tonsil MSCs in wound repair**

**Supplementary Methods**

**Immunostaining**

Mouse TNF-α and IL-10 were detected by immunohistochemistry. Mice were sacrificed and wound beds were excised, fixed in 4% paraformaldehyde followed by consecutive tissue processing steps and embedding in paraffin. Sections of 4 μm thickness were prepared and stained with anti-CD31, anti- TNF-α and anti-IL-10 antibodies (Abcam, Cambridge, UK). Slides were then incubated with biotinylated antibodies for 30 min, followed by the incubation with streptavidin HRP for 30 min. Positively stained cells were visualized by incubating the slides with 3,3-diaminobenzidine (DAB) for 5–10 min. Slides were washed with distilled water followed by the counterstaining with hematoxylin. For negative controls, tissues without primary antibodies were used.

**Preparation of Conditioned Media from TMSCs**

To harvest conditioned media of TMSCs for in vivo efficacy assessment, TMSCs (3 × 10^5^/well) were seeded in 6-well plates, and after 24 hours, TMSCs were washed 5 times and fresh α-MEM was added. After 3 days, the media was harvested and after centrifugation and filtration through a 0.22 μm syringe filter, conditioned media was topically applied into wound beds. Fresh α-MEM was used as vehicle control.

**Supplementary Figure**


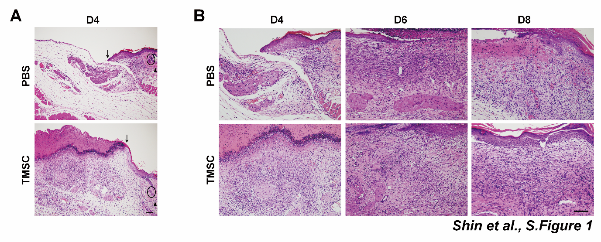


**Figure S1. Detailed demonstration of histopathology.**

(A-B) Skin wound lesions were collected and processed for H&E staining to perform histological assessment. (A) Representative H&E stained sections are shown. The epithelial margins and the wound edges are indicated by arrows and arrowheads, respectively. The skin appendages are located in the circle. (B) Representative H&E stained sections with higher magnitude are demonstrated. (day 4, 6 and 8). Scale bar=200 μm.


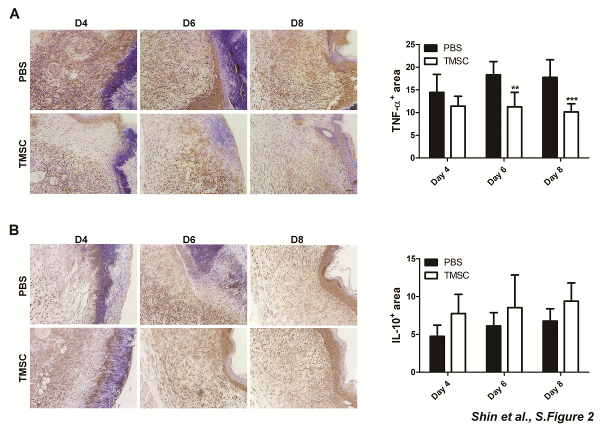


**Figure S2. Immunostaining of cytokines**

(A-B) Skin wound lesions on day 4, 6 and 8 were collected and processed for immunostaining to assess the infiltration of immune cells producing cytokines. (A) TNF-α^+^ cells or (B) IL-10^+^ cells were stained and visualized by DAB staining. Stained area was quantified using Image J. ** *P*<0.01, *** *P*<0.001. Results are shown as mean ± SD.


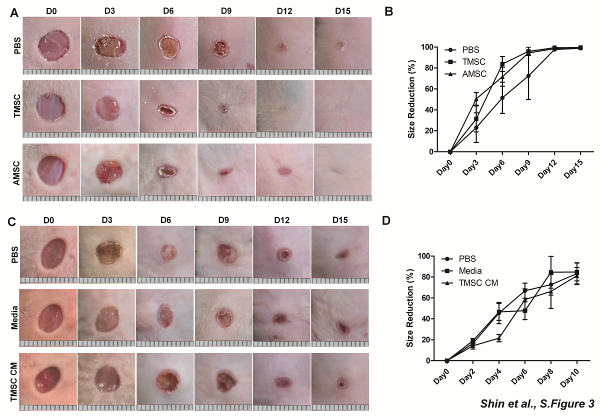


**Figure S3. Efficacy of TMSCs in wound model compared with AMSCs or TMSC CM**

(A) Representative time-course images of wound closure in a murine excisional skin wound model after TMSCs or ASCs administration. (B) The reduced wound size (%) relative to day 0 lesion (0%; complete wound closure is considered as 100%) was determined. (C) Representative time-course images of wound closure in a murine wound model after TMSC CM or Media (vehicle control) administration. (D) The reduced wound size was determined. Results are shown as mean ± SD.
